# What will it take to bring LAED medication regimens to young people?

**DOI:** 10.1002/jia2.26098

**Published:** 2023-07-13

**Authors:** Sybil Hosek, Lynda Stranix‐Chibanda

**Affiliations:** ^1^ Departments of Psychiatry & Infectious Disease Stroger Hospital of Cook County Chicago Illinois USA; ^2^ Child and Adolescent Health Unit Faculty of Medicine and Health Sciences University of Zimbabwe Harare Zimbabwe

1

Two long‐acting extended delivery (LAED) antiretrovirals for HIV (cabotegravir/rilpivirine for treatment and cabotegravir alone for prevention) were recently licensed in adolescents around the same time as adults (Table 1). We commend the early inclusion of minors in product development trials, reflecting many years of advocacy and evolving ethical standards. However, regulatory approval allowing new product use in adolescents is a necessary but insufficient condition for equitable implementation. Interventions aimed at the socio‐ecological barriers to engaging with HIV treatment and prevention services (e.g. stigma, parental disclosure, medical mistrust and healthcare access) must first be deployed to fulfil expectations that LAED products will be a gamechanger, transforming HIV outcomes in young key populations.

**Table 1:** Long‐acting and extended delivery products for HIV prevention and treatment

Product	Regimen	Regulatory timeline
**HIV TREATMENT**		
Cabotegravir/rilpivirine	Intramuscular injections either monthly or every 2 months after a loading dose	October 2020—EMA: recommended rilpivirine and cabotegravir to be used together for the treatment of HIV in adults. January 2021—FDA: approved in adults as a complete regimen for the treatment of HIV. March 2022—FDA: approved in adolescents aged 12 years and over weighing at least 35 kg.
**HIV PREVENTION**		
Dapivirine vaginal ring	Monthly insertion	July 2020—EMA: provided a positive benefit–risk opinion for use in adult women as a complementary prevention approach in addition to safer sex practices when women cannot use or do not have access to oral oral PrEP. January 2021—WHO: adolescent use approved simultaneously with adults; may be offered as an additional HIV prevention choice for women at substantial risk of HIV infection as part of a combination of prevention approaches. From July 2021—various country drug regulatory authorities: approved for use in adult women. Some countries allow use in adolescents.
Cabotegravir	Intramuscular injections every 2 months after a loading dose	December 2021—FDA: approved simultaneously in adolescents and adults weighing at least 35 kg. July 2022—WHO: adolescent use approved simultaneously with adults; may be offered as an additional HIV prevention option for people at substantial risk of HIV infection as part of combination prevention approaches.
Lenacapavir	Subcutaneous injections every 6 months	Phase 3 trials ongoing (NCT: 04925752 & 04994509)

Abbreviations: EMA, European Medicines Agency; FDA, US Food and Drug Administration; PrEP, pre‐exposure prophylaxis; WHO, World Health Organization.

Young people bear a disproportionate burden of HIV globally yet continue to have the worst outcomes in both treatment and prevention cascades [1]. Compared to all populations living with HIV, adolescents aged 13–24 are the age group most likely to not know their status or access antiretroviral treatment (ART), have higher levels of skipped medication doses and missed medical visits, and demonstrate the lowest rates of viral suppression even in the context of widespread access to ART [2, 3].

Addressing the slow rate of decline in new HIV infections among key populations of youth remains critical to controlling the HIV pandemic, especially in adolescent girls and young women in Africa and sexual and gender minority youth globally. Despite global guidelines recommending tenofovir‐based oral pre‐exposure prophylaxis (PrEP) for adolescents at substantial risk, HIV prevention efforts are severely undermined by profound inequalities in PrEP access for key youth populations [4]. For example, only 11% of youth with a PrEP indication in the United States are taking it [2]. A significant proportion of youth do not persist with oral PrEP, and strategies are needed to help youth assess their ongoing risk of acquiring HIV, remain motivated and overcome adherence challenges for daily oral PrEP [5].

LAED products offer tremendous promise to curb the challenges of medication adherence by simplifying regimens and decreasing daily pill burdens. LAED products also provide an increased level of privacy that can ameliorate concerns regarding stigma and unintentional disclosure associated with sexual activity and/or sexual identity for PrEP users. The availability of safe, efficacious LAED options for youth continues to grow and expands the choices youth have for products that fit their needs and circumstances (see Table 1). Unfortunately, significant barriers exist that interfere with the proper implementation and subsequent access to these products.

Worldwide, access to quality sexual and reproductive health, including HIV testing, treatment and prevention, is a challenge for youth. Less than optimal HIV testing rates can be attributed, in part, to the stigmatization of HIV as well as biased perceptions of sexual activity. Stigma and discrimination can have profound negative impacts on the health of young key populations. For example, people living with HIV are twice as likely to significantly delay engagement in care if they perceive high levels of stigma and discrimination regarding their HIV status [6]. To reduce HIV‐related stigma and encourage the uptake of new LAED HIV prevention tools, a status‐neutral approach is needed in which HIV testing is the natural first step towards engagement with HIV services, for either prevention or treatment [7]. Routinization of HIV testing in schools, communities and households can decrease stigma and discrimination towards key populations living with and at risk for HIV.

Adolescent sexual activity remains taboo in some communities, affecting service providers’ attitudes towards providing sexual and reproductive health services, including PrEP, to sexually active adolescents. In the case of sexual and gender minority youth, overt hostility directed towards the community blocks access to HIV prevention, treatment and other health services [8, 9]. The provision of effective resources to community‐led advocacy and health organizations is critical for engaging key populations in essential prevention and treatment research and services and overcoming barriers [10].

Worldwide, laws that inhibit the autonomy and decision‐making abilities of youth (including young pregnant women) make it increasingly difficult if not impossible to access essential sexual and reproductive health services. The Joint United Nations Programme on HIV/AIDS (UNAIDS) reports that among 141 countries with available data, 75% require adolescents to obtain parental/guardian consent for HIV testing [11]. Similar rates of restrictions related to contraception are evident, meaning that in many of these countries, adolescent girls can legally engage in sex but not access HIV testing or contraceptive services. The debate also swirls around the autonomy of pregnant women to make decisions about their health and safety as well as that of the developing foetus [12, 13]. Whether it is the ability to consent for trial participation, to make choices regarding medications during pregnancy or to make decisions about pregnancy termination, young women's autonomy continues to be questioned by policymakers and regulatory authorities. Finally, attacks on the autonomy of medical providers in countries like the United States to make decisions with their patients around sexual and reproductive health threaten to upend HIV prevention progress among key populations. Whether it is abortion prohibition or legal interference with the provision of gender‐affirming care, healthcare providers are at risk of losing their autonomy to practice without fear of legal ramifications [14]. To reach the full potential of LAED regimens, respect for the decision‐making autonomy of patients and providers is essential.

In sum, the implementation of wholistic HIV prevention and treatment services is much more than administering a biomedical product, *regardless of whether the product is LAED or not*. Integrated strategies must incorporate social and structural components to provide the support young people (and their parents/guardians) need for effective and sustained sexual and reproductive health. Almost 20 years ago, the World Health Organization issued recommendations for HIV services to be based around five adolescent‐friendly principles: accessible, acceptable, equitable, appropriate and effective [15]. However, the persistent disparities in HIV outcomes among youth indicate these principles must be broadly applied to support the development, effective implementation and equitable delivery of LAED products for HIV prevention and treatment.

## COMPETING INTERESTS

LS‐C received travel support from ViiV to attend the International Workshop on HIV and Adolescence 2022, Cape Town, South Africa. SH has no conflicts to declare.

## AUTHORS’ CONTRIBUTIONS

SH and LSC contributed equally to researching, writing and editing this manuscript.
